# Attention Allocation for Dysphoric Information in Adults with Depression Symptoms Using Eye-tracking and Mouse-tracking

**DOI:** 10.1371/journal.pone.0318923

**Published:** 2025-04-07

**Authors:** Mary E. McNamara, Jason Shumake, Christopher G. Beevers

**Affiliations:** 1 Department of Psychology and Institute for Mental Health Research, University of Texas at Austin, Austin, Texas, United States of America,; 2 McLean Hospital, 115 Mill St, Belmont; University of Pavia: Universita degli Studi di Pavia, ITALY

## Abstract

Biased attention for dysphoric stimuli is thought to maintain depression, but poor measurement has limited prior tests of this hypothesis. The current study examined the association between biased attention for dysphoric information and depression using a novel free viewing attention bias task combined with measuring line of visual gaze via eye tracking or a behavioral proxy for line of visual gaze via mouse tracking in three samples of college students using in-person eye-tracking (Experiment 1, *N* =  129) and remotely collected mouse-tracking (Experiment 2, *N* =  79; Experiment 3, *N* =  154). Mixed effects regression analyses revealed that depression severity was significantly associated with greater attention for dysphoric stimuli in Experiments 1 and 2, but not Experiment 3. Results suggest that depression severity is associated with attention for dysphoric information (although findings from Experiment 3 temper this conclusion) and that eye- and mouse-tracking may be good options for measuring attention bias in depression. Additional work using longitudinal research designs seems warranted to further examine the relationship between attention bias for dysphoric information and the maintenance of depression.

## Introduction

Attention bias refers to an information processing bias central to Beck’s model of depression [1]. Briefly, attention bias is the tendency for depressed individuals to spend more time attending to mood-congruent, typically dysphoric, stimuli (both internal and external) and less time attending to positive stimuli than their non-depressed counterparts. Traditionally, these attentional processes have been examined using externally-directed (i.e., visual) attention for stimuli, namely words and facial expressions [2]. While non-depressed individuals generally exhibit an attentional preference away from negative information, depressed individuals tend to spend more time viewing dysphoric stimuli, relative to individuals reporting little-to-no depression symptoms [3–7]. Additionally, depressed individuals have difficulty disengaging their attention once captured by sad stimuli [8] and take longer to disengage than both never-depressed participants [9] and individuals with lower depression symptoms [10]. However, these effects have not always been consistent, and other studies have not captured evidence of attention bias in depression [11,12]; see [13] for a review of mixed evidence).

According to cognitive theory, these biased attentional processes will yield downstream effects: depressed persons will ultimately take in more negative information and less positive information about themselves, the world, and others than their non-depressed peers [1]. Importantly, these attentional biases can also function as part of a feedback loop, where depressed persons might also have increased awareness of their unwanted thoughts, feelings, and physiological symptoms, which may engender more dysphoria and extend negative moods [14], maintaining the cycle [15,16]. Indeed, both attention bias [17] and delayed disengagement from sad faces in turn predict difficulty with mood recovery [9].

However, the assessment of attention bias in depression has been infamously illusive and fraught with challenges, due in large part to two particular issues. The first and more widely discussed obstacle has been difficulty with reliable measurement and poor psychometric properties of the bias metrics. Traditionally, the most widely-used behavioral paradigm for measuring attention bias has been the dot-probe task [18]. In the dot probe, participants are presented with stimuli (most often words or faces) on either side of the screen, typically of neutral and emotional (e.g., dysphoric) valences. Both stimuli then disappear and are replaced by a dot in the former location of one of the two stimuli. Participants indicate with a keyboard press whether the dot appeared on the left or right side of the screen, and a bias score is derived from this reaction time data. The bias score is used to infer attentional bias: attentional preference for sad stimuli is presumed when participants exhibit shorter reaction times when the dot appears in the location of the sad stimuli (implying the participant was already looking there), and longer reaction times when the dot appears in the location of the neutral stimulus (suggesting the participant was looking in the location of the sad stimulus and needs to disengage and shift attention to the other side of the screen).

However, the psychometrics of the traditional reaction time bias score derived from the dot probe task are resoundingly quite poor [19]. In particular, the bias metric has repeatedly been found to be unreliable in terms of the test-retest reliability [20,21]. A recent evaluation of the threat version of the dot-probe (where participants are presented with threat and neutral stimuli) involving 9,600 participants found that, across 36 different variations of the task, none produced internal reliabilities sufficient to justify the use of the bias difference score [22]. Attempts have been made to improve the psychometrics through computational modeling [23], as well as the use of eye-tracking instead of reaction-time based data to compute the bias score from the dot probe [24]. Methodologically, eye-tracking is a more precise means of measuring participant gaze, as opposed to inferring it from reaction time data. Moreover, the components of the bias score are often heavily correlated when using reaction time data, which contributes to instability of the bias score [25,26]. Despite these efforts, there have been continued calls to develop more psychometrically sound assessments of attentional bias [27].

The second principle challenge is that behavioral approaches of measuring attention bias in depression are largely an artifact of history and may not be optimally designed for depression in particular. The dot-probe task was first used to assess attention bias in patients with clinical levels of anxiety (e.g., the aforementioned threat dot-probe), and the verbal stimuli used depicted words that corresponded to social or physical threats [18]. Attentional bias in anxiety is characterized by hypervigilance and rapid, initial orienting to threat, and the design of the dot-probe task was originally suited to capture this process as it manifests in anxiety. In contrast, attentional bias in depression is marked by more elaborative processing, such that when individuals’ attention is captured by dysphoric stimuli, they experience difficulty shifting their attention away [6,28]. Indeed, later research demonstrated that attentional bias effects in depression tend to emerge in the context of stimuli being presented for longer durations [2], suggesting that different paradigms may be needed to optimally measure attention bias as it manifests in depression. This led to the development of free-viewing tasks that allowed for more sustained processing of emotional information to better capture bias effects [4,29–31]. Importantly, in the face of poor psychometrics and mis-aligned task design, the ability to test the role of attention bias in cognitive theory has been significantly hampered.

Deriving metrics from eye-tracking data used alongside free-viewing tasks appears to produce better quality psychometrics than the dot-probe task, particularly when using extended dwell time over early orienting measures [32,33]. However, collecting eye-tracking data in-person is sometimes unfeasible (due, for instance, to expensive equipment or when trying to recruit participants from other geographic locations). In these instances, mouse-tracking can be paired alongside free-viewing tasks as a proxy for eye-tracking. Prior work has shown that mouse-tracking can measure attention with high validity and reliability [34]. Mouse-tracking functions by first occluding the stimuli on the screen using a Gaussian blurred overlay, mimicking peripheral vision. Next, participants use a clear, circular aperture that is controlled by moving their mouse to “view” stimuli, allowing researchers to track the amount of time spent “viewing” emotional versus neutral stimuli. Evaluating the dwell time from the mouse-tracking data revealed these metrics had good psychometrics [35], consistent with what others have found [36].

In the present study, we examined the role of attentional bias in depression using a gold-standard eye tracking method and a more novel mouse-tracking method. We used the same attention bias task with these approaches: a free-viewing task that uses a 4 x 4 matrix array of sad and neutral facial expressions [30,31]. Importantly, the metrics derived from these free-viewing tasks seem to have good psychometric properties [30]. Having first demonstrated that dwell time for facial stimuli had strong internal consistency and unidimensionality in both the eye-tracking version of the task [33], and the mouse-tracking version [35], we turn towards using these metrics to evaluate whether depressed individuals display an attentional bias for dysphoric stimuli (faces) across one eye-tracking and two mouse-tracking samples. To-date, no studies have used this free viewing paradigm with mouse-tracking among people with depression. The current study involved data collection from three samples. In Experiment 1, we collected data from *N* =  130 college students using an eye-tracking version of this free-viewing task. In Experiment 2, we tested a similar version of the task using mouse-tracking in an online sample of *N* =  79 college students. In Experiment 3, we attempted to replicate the findings of Study 2 in a larger sample (*N* =  154) of college students.

Using this free-viewing task, our aim was to determine whether attentional biases are observed among those with elevated depression, as predicted by Beck’s cognitive theory of depression. Specifically, we examined whether depression severity was associated with greater attention for dysphoric stimuli relative to neutral stimuli. We expected a significant depression severity by stimuli valence interaction, as higher depression severity was expected to be associated with more time spent viewing dysphoric stimuli compared to neutral stimuli. This work has important implications for obtaining assessments of attention bias remotely (thereby opening up the possibility for large-scale online research) and for testing an important aspect of the cognitive theory of depression.

## Methods and Procedures

### Experiment 1

#### Methods.

The procedures and methods for Experiment 1 are described in detail in a previous publication documenting the psychometrics of the data derived from this task [33], however, we describe our sample and recapitulate the important methodological details here briefly.

#### Participants.

We initially collected data from 138 college students who received course credit for their participation. We excluded 9 individuals due to issues completing the experiment (only completing one of the two blocks and/or technical malfunctions during the task administration), or missing more than 50% of their data. This left us with a sample of *N* =  129.

Participants were eligible for the study so long as they were (a) between the ages of 18-45 years old; (b) able to speak, read, and understand English fluently; and (c) willing and able to provide informed consent. The study was approved by the University of Texas Institutional Review Board and all participants gave written consent to participate through REDCap (Research Electronic Data Capture), a secure web application for building and managing online studies. Data collection began on 3/14/2019 and concluded on 12/4/2019, spanning the spring, summer, and fall academic semesters of 2019.

Descriptive characteristics can be found in Table 1. Average age was 19.4 (*SD* =  1.4). The sample was mostly women (55.8%), non-Hispanic (67.4%), and the most common race reported was white (48.8%).

**Table 1:** Participant Demographics.

Characteristic	N (%)	N (%)	N (%)
Sample	Study 1 (2019)	Study 2 (2021)	Study 3 (2022)
Format	Eye-tracking	Mouse-tracking	Mouse-tracking
Sample Size	*N* = 129	*N* = 79	*N* = 154
Age	M = 19.4 (SD = 1.4)	M = 19.3 (SD = 1.3)	M = 19.1 (SD = 1.4)
Reported Gender			
Woman	*n* = 72 (55.8%)	*n* = 40 (50.6%)	*n* = 92 (59.7%)
Man	*n* = 57 (44.2%)	*n* = 37 (46.9%)	*n* = 59 (38.3%)
Other	*n* = 0 (0.0%)	*n* = 2 (2.5%)	*n* = 3 (1.9%)
Never married	128 (99.2%)	76 (96.2%)	151 (98.1%)
Ethnicity			
Hispanic	42 (32.6%)	25 (31.6%)	56 (36.4%)
Non Hispanic	87 (67.4%)	54 (68.4%)	97 (63.0%
Missing	0	0	1 (0.65%)
Race			
Asian	38 (29.5%)	14 (17.7%)	35 (22.7%)
Black or African American	8 (6.2%)	11 (13.9%)	7 (4.5%)
American Indian/ Alaska Native	4 (3.1%)	0 (0.0%)	3 (1.9%)
Native Hawaiian or Pacific Islander	0 (0.0%)	1 (1.3%)	0 (0.0%)
White	63 (48.8%)	43 (54.4%)	86 (55.8%)
Multiracial	6 (4.7%)	3 (3.8%)	13 (8.4%)
Unknown/not reported	10 (7.8%)	7 (8.9%)	10 (6.5%)
Years of Education	M = 13.9 (SD = 1.1)	M = 13.0 (SD = 1.1)	M = 12.8 (SD = 1.0)
BDI-II (20 Item)	M = 9.1 (SD = 7.9)	—	—
PHQ-8	—	M = 11.2 (SD = 6.4)	M = 9.9 (SD = 5.8)
IDAS Dysphoria	—	M = 27.0 (SD = 10.3)	M = 24.3 (SD = 9.2)

#### Materials.

In this sample, depression severity was measured using the Beck Depression Inventory-II (BDI-II; [37]). In the present study, we used a 20-item version that excluded the suicidal ideation item. We removed the suicidal ideation item since immediate evaluation and triage would not be possible, as the experimental sessions were being conducted by undergraduate (e.g., unlicensed) research assistants. In this sample the 20-item BDI-II had strong internal consistency (ɑ = .91, 95% CI [.88,.93]).

### “Eye-Tracking Apparatus and Procedure

Eye-tracking was captured via a video-based eye tracker (EyeLink 1000 Plus Desktop Mount; SR Research, Osgoode, ON, Canada). Dominant eye for each participant was determined using a modified version of the near-far alignment task [38]. Calibration was completed prior to beginning the task in order to map the participants’ gaze onto the screen coordinates; a 13-point calibration routine was used. We allowed for natural head movement (e.g., we did not use a headrest), and used a head-based tracker to provide consistent eye-tracking. Sampling was taken at 250 Hz using the participant’s dominant eye.

The eye-tracking task was presented using a 23.6-inch CRT monitor (ViewPixx; VPixx Technologies, Quebec, Canada) with a screen resolution of 1920 × 1080 pixels (120 Hz refresh rate). The task was run using OpenSesame, a user-friendly graphical experiment builder, with options for scripting utilizing PsychoPy, open-source software for running experiments [39]. OpenSesame also has integration with Eyelink software for data acquisition. Data was initially processed using Eyelink Data Viewer.

#### Eye-tracking attention bias task.

In this study, we altered a previous version of a task originally designed by Lazarov and colleagues [30,31,40], which we have previously described in detail [33]. Matrices of sixteen faces in a 4 x 4 array are presented in each trial of this task, consistent with other free-viewing approaches that have been used in the literature for assessing more naturalistic patterns of visual attention [4].

Stimuli were chosen from the FACES dataset and were selected based on previously documented accuracy ratings [41]. In our task, we used equal numbers of middle-aged and young-adult actor images and did not use the older adult facial stimuli in an effort to be more consistent with the demographics of our participants.

**Fig 1:**
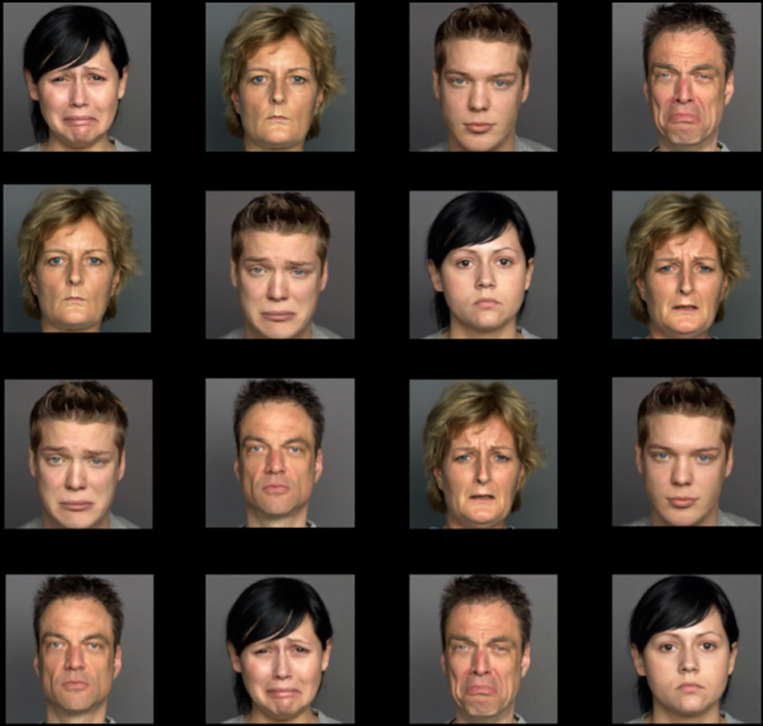
Example trial of the matrix task. Note: In our actual trials, we had the following constraints, consistent with Lazarov, Abend, and Bar-Haim (2016): (a) each actor could appear only once on the matrix, (b) there was an even split of genders in each matrix (8 male and 8 female), (c) there was an even split of valences in each matrix (e.g. 8 neutral and 8 sad), and (d) the four inner faces always contained two emotional and two neutral faces. This sample trial uses images approved for publication, so while criteria (b) - (d) are met, criterion (a) is not fulfilled in this example [41].

Each trial consisted of a 4 x 4 matrix of photographs on a black background. When designing the task, we selected 32 images of 8 male and 8 female actors, with neutral and sad faces for each (e.g., 8 sad male faces, 8 neutral male faces from the same actor, 8 sad female faces, 8 neutral female faces from the same actor). Only sad and neutral facial expressions were used in this task. We separated the images into four pools and generated 15 trials from each pool for a total of 60 trials. Consistent with the design of Lazarov and colleagues, the generation of the matrices were random with the following constraints: (a) each actor could appear only once on the matrix, (b) there was an even split of genders in each matrix (8 male and 8 female), (c) there was an even split of valences in each matrix (e.g., 8 neutral and 8 sad), and (d) the four inner faces always contained two emotional and two neutral faces. We refer to this task from herein as the matrix. An example trial can be found in Figure 1.

Our task consisted of 60 trials displayed for 10 seconds each, and trials were arranged in two blocks of 30 trials each. Between blocks, participants were allowed to take a break and were told to press the spacebar when they were ready to continue. Instructions for the task told participants to fixate on the fixation dot presented at the beginning of each trial. The following instructions were given for the task, “Before each matrix, a fixation dot will appear on the screen. Make sure to fixate on the dot when it appears. When the matrix appears, look at the images freely and naturally. Do you have any questions?” These instructions were used to allow for and encourage naturalistic processing of the images [30].

#### Eye Tracking Metrics.

From each stimulus presentation, we created 16 areas of interest (AOIs), one for each individual photograph presented in the image grid during each trial. We then collapsed the AOIs into two categories, one sad AOI and one neutral AOI for the corresponding faces (e.g., two AOIs per trial). We computed dwell time for sad and neutral faces, which in our prior work has had good internal consistency and unidimensionality [33]. Dwell time was operationalized as the sum of fixation durations across the course of the whole trial and is presented in milliseconds (ms).

#### Procedure.

Participants were recruited through SONA systems, a cloud-based recruitment and participant management system geared toward university research studies. This study was conducted by trained research assistants in the Mood Disorders Laboratory on-campus at the University of Texas at Austin. Participants completed the study in exchange for 1 hour of course credit.

Participants completed a consent form, followed by a demographic form and self-report questionnaires. They then completed the matrix task and were awarded course credit at the end of the experiment session.

## Experiment 2

### Methods

#### Participants.

We collected a sample of 108 college students from the University of Texas participant pool. However, before running our main analyses, we performed a number of data quality assessments typically performed when collecting data remotely and fully unsupervised, ultimately retaining a sample of *N* =  79. Each step of our data quality assessment is detailed in the section *Data Quality Analysis* below, along with the number of people excluded in each step.

Participants were eligible for the study so long as they were (a) between the ages of 18-45 years old; (b) able to speak, read, and understand English fluently; and (c) willing and able to provide informed consent. Participants were also required to use a desktop or laptop computer to access the study. This study was approved by the University of Texas Institutional Review Board. Since participants consented to the study online through clicking a checkbox, we obtained a waiver of consent documentation from the IRB. Data collection took place between 11/23/2021-12/13/2021.

Descriptive characteristics of the sample can be found in Table 1. Our sample was majority female (50.6%), white (54.4%), and non-Hispanic (68.4%). Average age was 19.3 (*SD* =  1.3) and mean PHQ-8 score was 11.2 (*SD* =  6.4), indicating mild depression severity.

### Materials

#### PHQ-8.

We measured depression severity via the Patient Health Questionnaire-8 (PHQ-8). The PHQ-8 is identical to the PHQ-9 except the suicide item has been removed. The use of the PHQ-8 is potentially advantageous to use in internet-based research where immediate follow-up for a risk evaluation is not logistically possible [42]. The PHQ-8 appears to have similar properties as the PHQ-9 [43], and one report found no significant differences in internal consistency and validity between the 8 and 9-item versions [44]. Internal consistency in our sample was similarly high (alpha = .91, 95% CI [.87,.94]).

#### IDAS Dysphoria.

As a test of robustness, in this study we included a secondary measure of depression severity, the Inventory of Depression and Anxiety Symptoms (IDAS) dysphoria subscale. The IDAS questionnaire was derived using factor analysis and the dysphoria subscale appears to have good psychometric properties [45,46] including unidimensionality justifying use of a total sum score. Internal consistency in our sample was also excellent (alpha = .94, 95% CI [.92,.96]).

#### Attentive Responding Scale - Infrequency.

Given that this study was being administered online, we incorporated a number of attention checks to ensure participants were engaged in the task. For data cleaning purposes, we used the 11-item Infrequency subscale of the *Attentive Responding Scale - 33* item version [47] (further information about all data cleaning procedures are detailed below in the Assessment of Data Quality section). The infrequency subscale is designed to capture participants with a highly unlikely pattern of responding. Items are designed to encourage highly skewed responses from most participants (e.g., “ My favorite subject is agronomy”, “ I love going to the DMV (Department of Motor Vehicles)”, “My main interests are coin collecting and interpretive dancing.”) Frequent endorsement of these items results in a larger score. Participants who score above a previously established threshold ( > 11.5 [47]) were excluded on the basis of inattentive responding.

#### Mouse-tracking attention bias task.

Our mouse-tracking task was very similar to the task described in sample one, with a few exceptions. The major difference was the number of trials and length of stimulus presentation. When piloting the task, we observed that it takes longer to navigate through all the images in the matrix using the mouse cursor than when simply navigating through visually with line of sight. Therefore, we doubled the presentation time of each trial to 20 seconds, and reduced the total number of trials to 30, distributed across two blocks (15 trials each). We reduced the number of trials so as not to increase the total time spent on the task and therefore increase participant burden.

Between blocks, participants were allowed to take a break and were told to press the spacebar when they were ready to continue. Instructions for the task read: “*In this task, you will use your mouse to look at the pictures. You will need to click on the fixation cross to make the pictures appear. You can then look freely at the pictures in any way you choose until they disappear. Press the spacebar when you are ready to continue with the task*.” The requirement to click the fixation cross at the beginning of each trial was implemented for engagement and to ensure participants started at the same point on the screen for each trial. In each trial, participants were presented with a 4 x 4 matrix of photographs; in this study, the images appeared on a white background.

**Fig 2:**
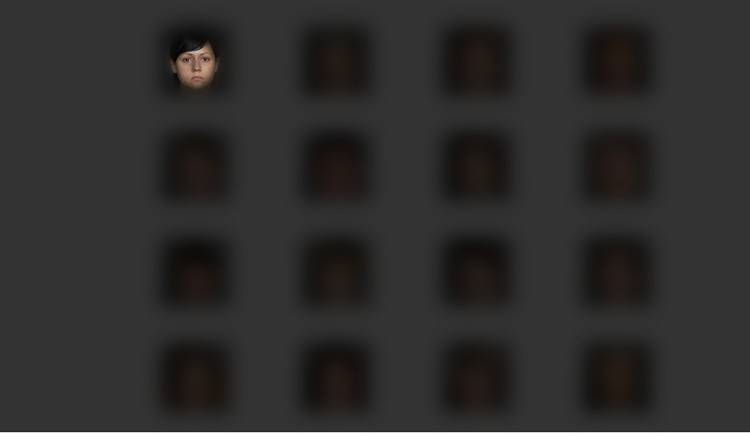
Example trial of matrix task with Mouseview.js when stimuli are occluded via Gaussian Blur, and the mouse-aperture is hovering over an image.

In combination with the task, we used Mouseview.js, a JavaScript library that is integrated with Gorilla experiment builder. The Mouseview software first utilizes a Gaussian blur overlay, a photo processing technique that blurs the images on the screen using a Gaussian function. The entire webpage is then blurred and all images on the screen are obfuscated. Next, the participant can view portions of the screen by moving a small circular aperture with the computer’s mouse. Participants are able to view the images only via hovering over the picture with the aperture associated with the location of the mouse. Mouseview then tracks the coordinates of the aperture viewing window to identify where and how long participants have been viewing stimuli. Participants also were instructed to look at the pictures by moving their mouse any way they chose until the pictures disappeared; all participants were given a practice trial first as a demonstration. An example trial of the free-viewing task presented with Mouseview can be found in Figure 2. A practice trial was given to participants as a demonstration. The Gaussian overlay can be adjusted in terms of color (e.g., black vs color overlay), opacity (how transparent the overlay is), and degree of blur. We used the default settings within Gorilla (color =  black; alpha (opacity) =  0.8; Gaussian blur =  20). We also maintained the default size for the viewing aperture (aperture size =  5% of computer screen).

#### Mouse Tracking Metrics.

Similar to the eye-tracking data described above, 16 AOIs were created from the 4x4 matrix in each trial. The AOIs were then collapsed into two omnibus categories per trial: one sad AOI and one neutral AOI for the corresponding faces. We computed total scan time for sad and neutral faces on a per trial basis. Scan time was operationalized as the sum of the amount of time the aperture was positioned over the faces across the course of the whole trial (presumably reflecting attention), and is presented in milliseconds (ms). We have previously established that the metrics used in this analysis have good psychometric properties [35].

#### Procedure.

Participants were recruited through SONA systems, a cloud-based recruitment and participant management system geared toward university research studies. Within SONA they were given a link to access the study hosted on Gorilla – an online platform for running behavioral experiments [36]. All aspects of this study were conducted through the Gorilla platform (e.g., consent, questionnaires, behavioral tasks).

Participants completed the study in exchange for 1 hour of course credit. While the study protocol was less than 1 hour, participants were given a 2-hour window from the time they initiated the study to finish the questionnaires and tasks (although some of the tasks had time limits). If participants were interrupted or were not aware of the 2-hour time limit and wanted to be able to complete the study for course credit, they had to restart the study from the beginning. No one was given more than 1 additional attempt at completing the study.

Participants completed a consent form at the beginning of the study. They were then randomized to one of two studies, the other of which is not described in this report; no participants completed both studies. Next, they filled out a demographic form and self-report questionnaires. They then completed the matrix task, along with other behavioral tasks not relevant to the current study. Finally, they completed a question asking how much effort they had put into the study (detailed further below) and were awarded course credit.

#### Assessment of Online Data Quality.

Given the importance of assessing participant attention and effort [47], particularly in online research [48,49], we incorporated a number of attention checks [50] into our data cleaning pipeline for our online samples prior to computing our results. First, we removed individuals who were missing 50% or more of their trials in the matrix task. Trials were also coded as missing if participants did not move the aperture over at least 4 faces in the trial (25% of the stimuli in the trial). Thus, if participants were engaging with less than a quarter of the stimuli per trial for 50% or more of the trials, we removed them on the basis of poor engagement (9 participants).

Next, we considered a measure of engagement, the Infrequency subscale [47]. Using a previously established cutoff score, we excluded 3 individuals who scored a 11.5 or higher on the ARS Infrequency scale, designed to capture a pattern of highly unusual responses, signaling inattention to the task (further details on this measure can be found in the materials section). One additional individual was excluded for failing to complete self-report data.

At the end of the experiment, we asked participants to rate their overall level of engagement: “Please answer the following question honestly. **You will still get credit for your participation, regardless of your answer.** How much effort did you put into the tasks in this study?” Participants could select the following responses: 1 - “A lot; I tried my best the whole time”; 2 - “Some; I gave it a shot”; 3 - “Not much; I was distracted”; 4 - “None; I clicked through randomly.” We then excluded 3 individuals that indicated a 3 or 4 on this item, self-disclosing that they had been distracted during the experiment.

At this point, we had a sample of 92 participants. However, after data collection was completed, the Gorilla software documentation was updated to warn experimenters that the Gaussian Blur component of Mouseview does not work as intended in Safari Browsers. Therefore, we had to exclude an additional 13 participants who had accessed the experiment on a Safari browser for a sample size of *N* =  79. Note that the same individuals may have met criteria for more than one data quality exclusion criterion. This level of attrition (108 - 79/ 108 =  26.8%) for remotely conducted studies is typical [50–52].

### Experiment 3

#### Methods.

The procedures for experiment 3 are identical to those in experiment 2. Because Gorilla is an online behavioral software platform for conducting experiments and our entire study was housed on Gorilla’s platform, we were able to simply make a copy of the project used for experiment 2 and initiate recruitment, which is ideal for a replication study. The only change that was made was to require participants use Chrome or Firefox as their browser for the experiment to ensure the Gaussian blur overlay would work as intended (described above in the Assessment of Data Quality section for Study 2). Thus, we only report on participant demographics, the internal consistency of the depression measures in the new sample, and our assessment of data quality in this methods section, as our materials, behavioral task, and data analysis protocols were the same as above.

### Participants

We initially collected a sample of 176 college students from the University of Texas participant pool. We once again performed a data quality assessment, utilizing the same data cleaning pipeline found in Study 2, ultimately retaining a sample of *N* =  154. Further information about our Data Quality Analysis and the number of participants excluded at each step can be found below.

We retained the same eligibility criteria as experiment 2: to be eligible, participants must be: (a) between the ages of 18-45 years old; (b) able to speak, read, and understand English fluently; and (c) willing and able to provide informed consent. Participants also needed a desktop or laptop computer to access the study. This study was approved by the University of Texas Institutional Review Board. Since participants consented to the study online through clicking a checkbox, we obtained a waiver of consent documentation from the IRB. Data collection took place between 8/30/2022-12/7/2022.

Descriptive characteristics of the sample can be found in Table 1. Our sample was majority female (58.7%), white (55.8%), and non-Hispanic (63.0%). Average age was 19.1 (*SD* =  1.4) and mean PHQ-8 score was 9.9 (*SD* =  5.8), indicating mild depression severity.

### Materials

#### PHQ-8.

Internal consistency for the PHQ-8 in this sample was (alpha = .91, 95% CI [.88,.93]).

#### IDAS Dysphoria.

Internal consistency for the IDAS dysphoria scale was (alpha =.91, 95% CI [.89,.93]).

#### Assessment of Online Data quality.

Again, we used the same data quality evaluation procedure described above for Study 2, including the same code in our scripts. First, we removed individuals who were missing 50% or more of their trials in the matrix task, removing 14 participants in this sample. We excluded an additional 4 participants for inattention, operationalized as scoring higher than 11.5 on the ARS Infrequency Scale. We excluded data from an additional 4 participants for indicating they had been distracted during experiment on the effort check item. Finally, because we required that participants not use Safari for this second iteration of the mouse-tracking study, we did not need to exclude as we did in the prior data cleaning pipeline. Thus, we retained a sample of *N* =  154, and an attrition level of only 12.5% (176 - 154/ 176 =  12.5%).

#### Sample Size Justification.

A detailed report of our power analyses can be found in supplementary materials (“sample-size-justification.pdf” at https://doi.org/10.18738/T8/RIV4X9). In summary, we used a simulation approach with the R package SIMR (Green & MacLeod, 2016) to estimate statistical power for the depression severity by stimuli valence interaction in a mixed effects model used in our analyses. We tested a range of effect sizes for the focal fixed effect interaction while keeping other simulation parameters (obtained from sample 1 and sample 2, respectively) constant.

As can be seen in supplementary materials, a sample size of 75 participants would be sufficient to detect a relatively small (*d* = .05) valence by depression severity interaction effect size using a random intercepts model with standard deviations for the random intercepts, fixed effects, residual variance, random intercept and slope variance-covariance matrix similar to those observed in Study 1. Thus, we have appropriate statistical power to detect a relatively small depression severity by stimuli valence interaction in Experiment 1 (*N* =  129).

Not surprisingly, sample size requirements are more demanding for the mouse tracking method given that data is collected in less controlled environments which should increase random error. Power analyses suggest that for our mouse tracking study, a sample size of about 200 would be sufficient to detect a relatively small (*d* = .05) valence x depression severity interaction effect size using a random intercepts model with standard deviations for the random intercepts, fixed effects, residual variance, random intercept and slope variance-covariance matrix similar to those observed in our Study 2. Thus, Studies 2 (*N* =  79) and 3 (*N* =  154) may have been underpowered to detect relatively small interaction effect sizes based on these power analyses, but statistical power to detect a larger (i.e., *d* = .08) depression severity x stimuli valence effect would be considered adequate.

### Data

#### Assessment of internal consistency.

We calculated and reported both alpha and omega total for each metric using the *psych* package in R [53]. We report both in response to calls to examine multiple metrics of internal consistency beyond Cronbach’s alpha [54].

#### Data Analysis.

Our data analysis approach was the same across studies and reused the same segments of code for consistency. Data were analyzed in R (version 4.4.0). We used the *tidyverse* packages for data cleaning and processing [55], along with *knitr* [56], *gridExtra* [57], and *ggpubr* [58]. We also used *itrak*, a package developed in-house for processing eye-tracking data [59], and adapted it our application for use with the Mouseview data. Mixed effect models were created using *lmerTest* [60], which uses lme4 [61] to run the models and then calculates degrees of freedom using the Satterthwaite method, along with significance tests for the coefficients. Assumptions were tested using the *performance* package [62]. Plots of the models were made with *sjPlot* [63], *sjmisc* [64], and *ggplot2* [65]. Tables of model coefficients were created using *rempsych* [66]. We calculated the internal consistency of the depression measures in our sample (IDAS dysphoria and PHQ-8) using *ltm* [67]. All code and data used in the current studies are available within the Mood Disorders Laboratory’s dataverse, hosted here: https://doi.org/10.18738/T8/RIV4X9.

#### Statistical Model.

We ran linear mixed-effects models predicting the amount of stimuli viewing time (dwell time (in milliseconds) for eye tracking, experiment 1, and scan time (in milliseconds) for mouse-tracking, experiments 2 and 3) - the amount of time participants spent looking at stimuli. In mixed effects regression models, we examined the interaction between the fixed effects of stimuli valence and participant depression score. Random effects for participant were also modeled (id). Initially, we attempted to include a random effect for both stimulus presentation (ia_id) and trial with an interaction of stimuli valence and participant depression, but this model was too complex to fit the data (i.e., the model would not converge). We then iterated through the following options for random effects, in order: 1. (1 | ia_id) +  (trial|id); 2. (1 | ia_id) +  (trial||id); 3. (1 | ia_id) +  (1 | id); 4. (trial|id); 5. (trial||id); 6. (1 | id). Only the last model using the random effect of id converged, so we included trial as a fixed effect. Thus, our model specification was:


$$\text{SD}=\frac{(C_{S}-C_{B})\times \text{F}\times V}{C_{M}\times A}$$

## Results

### Internal consistency of metrics

We examined the internal consistency of each of our dwell/scan time metrics. While the eye-tracking data metric had the strongest internal consistency (alphas = .95 for both sad and neutral dwell time), the mouse-tracking data also showed strong psychometrics (alphas between.80 -.86). Full description of internal consistency indices can be found in Table 2.

**Table 2:** Internal Consistency Indices.

Bias Metric	Alpha	Omega Total
*Study 1: Eye-tracking (n = 125)*		
Dwell time for sad faces	.95	.96
Dwell time for neutral faces	.95	.95
*Study 2: Mouse-tracking (n = 61)*		
Scan time for sad faces	.80	.85
Scan time for neutral faces	.85	.89
*Study 3: Mouse-tracking (n = 125)*		
Scan time for sad faces	.84	.87
Scan time for neutral faces	.86	.89

Note: Sample sizes in this table refer to the sample size the psychometric analyses were conducted on. Because nearly no data can be missing to compute the factor structure analyses, we had to reduce our data set to a smaller sample size to compute these metrics. These numbers represent individuals who were missing no more than 2 trials. Our mixed effects models and demographics were calculated based on the full sample.

### Tests of Attentional Bias in Depression

#### Experiment 1 (Eye-tracking)

**Fig 3:**
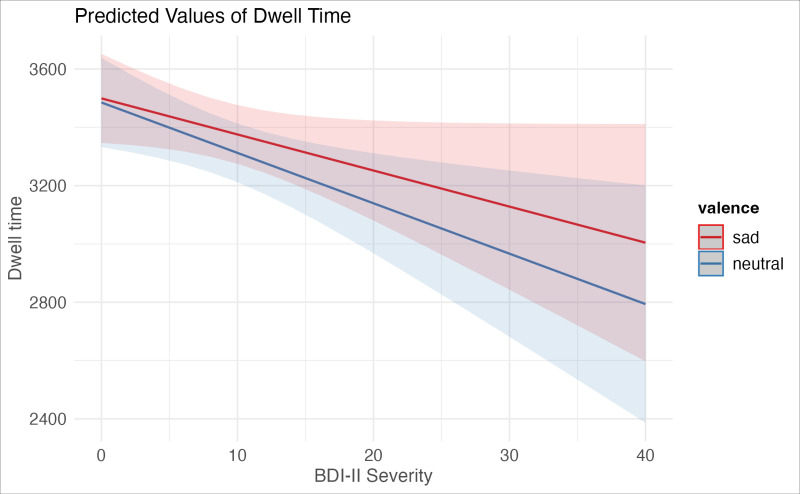
Significant Interaction of depression severity (BDI-II) and stimuli valence on dwell time.

*BDI-II:* Mixed effects model results for the first sample can be found in Table 3. The amount of time participants spent fixating on faces was significantly predicted by the interaction of BDI-II score and stimuli valence (β =  -4.915, t =  -2.099, p = .036). Depression yielded a steeper slope of reduced dwell times for sad faces relative to neutral; in other words, at higher levels of depression, dwell time for sad stimuli was greater than dwell time for neutral stimuli. A plot of these effects can be found in Figure 3.

**Table 3:** Experiment 1: Mixed effects model predicting Dwell Time (ms) from depression severity (BDI-II), stimuli valence, and trial.

Term	Estimate	Std. Error	*df*	t value	Pr(>|t|)
(Intercept)	3677.148	79.531	146.689	46.236	<.001
Depression severity	-12.374	6.510	135.604	-1.901	0.059
Neutral stimuli valence	-14.065	28.069	15281.978	-0.501	0.616
trial	-6.044	0.531	15282.746	-11.391	<.001
Depression severity x neutral stimuli valence	-4.915	2.342	15281.978	-2.099	0.036

Note: Depression severity was measured using a 20-item version of the BDI-II

### Experiment 2 (Mouse-tracking)

#### PHQ-8.

**Fig 4:**
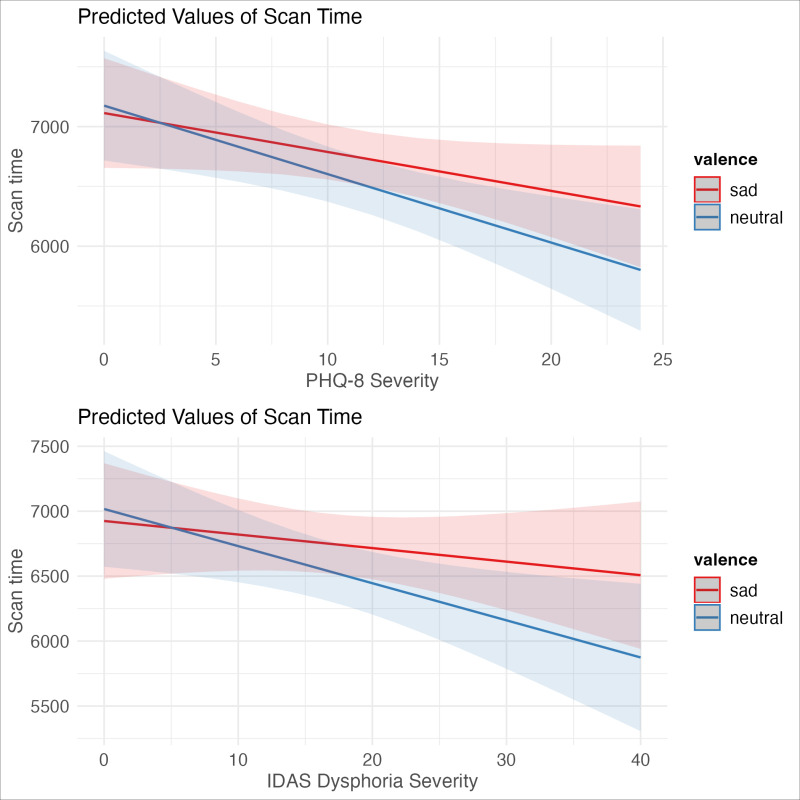
Interaction of depression severity and stimuli valence when using PHQ-8 (4A, marginally significant) and IDAS Dysphoria subscale (4B, significant).

Results of the model using PHQ-8 as the measure of depression severity can be found in Table 4. The amount of time spent scanning faces was again predicted by the interaction of depression severity and image valence, although the results did not reach significance (β =  -24.715, t =  -1.91, p = .056). These results were in the same direction as the pattern seen in study 1 where depression reduced scan times for both face image types, but produced a shallower reduction in sad face. A plot of these effects can be found in Figure 4A. In our sensitivity analyses (which can be found in the Supplementary Materials file “depressionbyvalence_paper.pdf” at https://doi.org/10.18738/T8/RIV4X9), we also observed a three-way interaction of depression severity, stimuli valence, and trial (β =  -6.25, t =  -4.16, p < .001). Individuals with greater depression severity spent more time viewing sad stimuli than neutral stimuli as the task progressed, whereas individuals with lower depression severity spent less time viewing sad stimuli and more time viewing neutral stimuli as the task progressed.

**Table 4:** Experiment 2: Mixed effects model predicting Dwell Time (ms) from depression severity (PHQ-8), neutral stimuli valence, and trial.

Term	Estimate	Std. Error	*df*	t value	Pr(>|t|)
(Intercept)	7121.676	243.607	115.152	29.234	<.001
Depression severity	-32.538	18.160	98.893	-1.792	0.076
Neutral stimuli valence	62.001	165.547	4335.303	0.375	0.708
trial	-0.603	4.779	4353.381	-0.126	0.900
Depression severity * neutral stimuli valence	-24.716	12.939	4335.308	-1.910	0.056

Note: Depression severity was measured using the PHQ-8

#### IDAS Dysphoria Subscale.

As a test of robustness, we also created an identical model using the IDAS Dysphoria subscale instead of the PHQ-8. Results of this mixed effects model can be found in Table 5. The amount of time participants spent scanning faces using mouse-tracking was significantly predicted by the interaction of depression severity and stimuli valence (β =  -18.145, t =  -2.273, p = .023). Individuals with higher depression scores spent less time viewing faces overall, but there was less of a reduction in viewing time for sad faces relative to neutral (Figure 4B). As with the PHQ-8 data, these results were explained by a higher-order 3-way interaction in our sensitivity analyses of depression, stimuli valence, and trial (β =  -3.62, t =  -3.92, p = .000). Individuals with elevated depression symptoms spent more time viewing sad faces over neutral as the task progresses. Participants endorsing fewer depression symptoms spent more time viewing neutral faces and less time on sad faces as the task progressed.

**Table 5:** Experiment 2: Mixed effects model predicting Dwell Time (ms) from depression severity (IDAS dysphoria subscale), neutral stimuli valence, and trial.

Term	Estimate	Std. Error	*df*	t value	Pr(>|t|)
(Intercept)	6933.085	236.934	114.343	29.262	<.001
Depression severity	-10.433	11.467	96.971	-0.910	0.365
Neutral stimuli valence	92.350	157.250	4335.091	0.587	0.557
trial	-0.595	4.778	4352.568	-0.125	0.901
Depression severity * neutral stimuli valence	-18.145	7.981	4335.097	-2.273	0.023

Note: Depression severity was measured using the IDAS dysphoria subscale

### Experiment 3 (Mouse-tracking)

#### PHQ-8.

**Fig 5:**
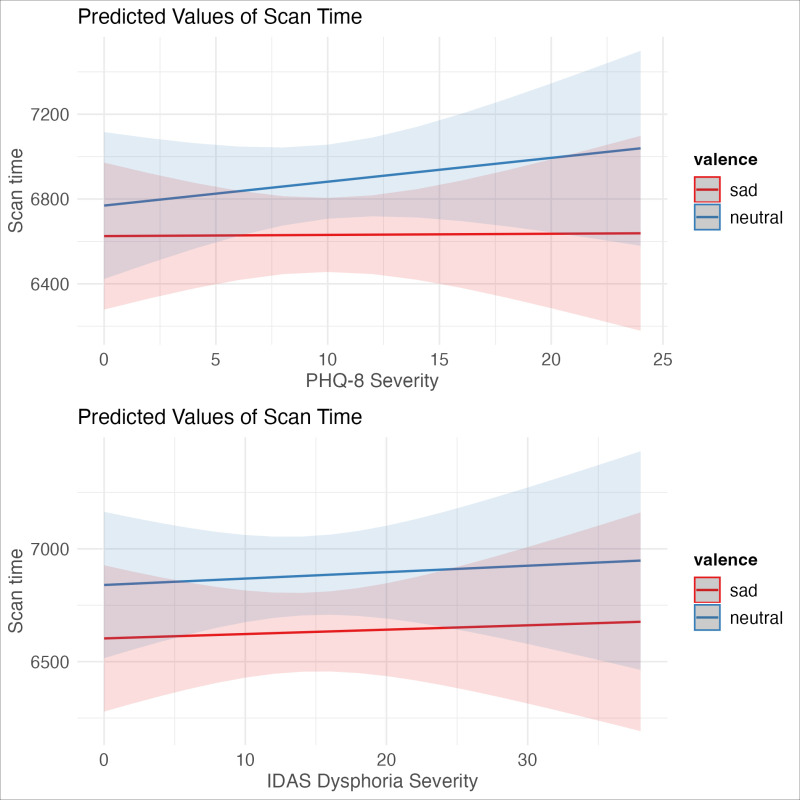
No interaction of depression severity and stimuli valence when using PHQ-8 (5A) or IDAS Dysphoria subscale (5B).

The mouse-tracking replication study diverged from the pattern of results in the other samples (Table 6). The interaction of depression severity and stimuli valence did not significantly predict scan time in the task (β = 10.706, t =  1.07, p = .285, see Figure 5A). After removing the interaction term, there was a main effect of valence, such that participants spent more time looking at neutral faces across the range of depression severity (β =  249.870, t =  4.29, p = .000), and a main effect of trial, such that scan time dropped as the task progressed (β =  -21.40, t =  -6.261, p = .000). This was explained by a higher order interaction of valence by trial in the sensitivity analyses (β =  13.756, t =  2.028, p = .0426), where participants spend less time viewing stimuli as the task progressed, but especially sad stimuli. All follow-up analyses can be found in the “depressionbyvalence_paper.pdf” document at https://doi.org/10.18738/T8/RIV4X9.

**Table 6:** Experiment 3: Mixed effects model predicting Dwell Time (ms) from depression severity (PHQ-8), neutral stimuli valence, and trial.

Term	Estimate	Std. Error	*df*	t value	Pr(>|t|)
(Intercept)	6927.494	183.175	216.338	37.819	<.001
Depression severity	0.549	15.408	187.659	0.036	0.972
Neutral stimuli valence	143.960	114.840	8389.213	1.254	0.210
trial	-21.399	3.418	8432.833	-6.261	<.001
Depression severity x neutral stimuli valence	10.706	10.004	8389.203	1.070	0.285

Note: Depression severity was measured using the PHQ-8

#### IDAS Dysphoria.

We again failed to replicate the finding that depression severity was associated with greater scan time for sad than neutral faces (β =  0.907, t =  0.142, p = .887, Table 7, see Figure 5B). After removing the interaction term, there was again a main effect of valence (β = 249.870, t =  4.29, p = .000), such that participants spent more time viewing neutral than sad stimuli, regardless of depression severity. There was also a significant main effect of trial, demonstrating that participants spent less time viewing stimuli as the task progressed (β =  -21.40, t =  -6.262, p = .000). This was again explained by a higher-order interaction of valence and trial (β =  13.743, t =  2.026, p = .0428), where participants viewed stimuli less over the course of the trial, particularly the sad stimuli.

**Table 7:** Experiment 3: Mixed effects model predicting Dwell Time (ms) from depression severity (IDAS dysphoria subscale), neutral stimuli valence, and trial.

Term	Estimate	Std. Error	*df*	t value	Pr(>|t|)
(Intercept)	6905.304	172.246	222.446	40.090	<.001
Depression severity	1.940	9.741	189.280	0.199	0.842
Neutral stimuli valence	236.884	108.226	8389.228	2.189	0.029
trial	-21.401	3.418	8432.820	-6.261	<.001
Depression severity x neutral stimuli valence	0.907	6.371	8389.229	0.142	0.887

Note: Depression severity was measured using the IDAS dysphoria subscale

We also conducted sensitivity analyses for each of the three samples examining the effect of trial, gender, and time of the academic semester (these analyses can be found in the Supplementary Materials). No consistent pattern of effect arose across samples.

## Discussion

The putative role of attention bias for dysphoric information in depression has been challenging to characterize, in part because of poor measurement. Thus, we used a psychometrically robust, free-viewing task designed to capture the elaborative processing of complex emotional stimuli configurations using eye-tracking and mouse-tracking approaches, which more directly measure line of visual gaze rather than inferring attention from reaction time. We examined whether attentional bias for dysphoric information was associated with depression severity in three samples (in-person with eye tracking, *N* =  129, remotely with mouse-tracking, *N* =  79 and *N* =  154). The results in studies 1 and 2 generally supported a depression-by-valence interaction, such that people with greater depression severity looked longer at sad faces relative to neutral faces compared to people with fewer depression symptoms. These results did not replicate in Study 3.

These findings are consistent with other studies that examine dwell time collected via eye-tracking-based free-viewing tasks. Individuals with elevated depression symptoms tend to spend more time looking at sad and dysphoric stimuli relative to neutral or happy faces [7], which is consistent with what we found in our eye-tracking study and the first mouse-tracking sample. Specifically, in regard to the matrix task, our results are mostly consistent with previous work. When using a happy/sad facial expression version of the matrix with eye-tracking, Lazarov et al. [30] found a significant group-by-valence interaction for average dwell time for individuals with clinical depression, high depression symptoms, and low depression symptoms. While all groups spent more time looking at happy faces relative to sad, the clinically depressed and high-depression severity groups spent significantly more time looking at sad faces than did the low-depression participants [30]. Klawohn et al. [31] also found evidence of a stimuli valence by group interaction (depressed participants versus healthy controls). Healthy control participants spent more time looking at neutral faces than sad, while depressed participants did not display this preference and allocated their attention evenly. The authors also reported that follow-up analyses confirmed depressed participants also viewed sad faces for longer periods than healthy control participants did [31].

Finally, Basel et al. [68] compared the performance of low-depression and high-depression participants across happy-neutral, sad-neutral, and sad-happy blocks of trials in their administration of the matrix task. They collapsed areas of interest (AOIs) into relatively negative and relatively positive stimuli. The valence of stimuli was determined by what it was paired with in each block of the task. For instance, neutral stimuli were considered to be negative stimuli when paired with happy stimuli but neutral stimuli were considered to be positive stimuli when paired with sad stimuli (hence the term “relatively” negative and “relatively” positive). Using this approach, Basel and colleagues reported a significant group-by-AOI-type interaction where low-depression individuals displayed a relatively positive bias, meaning participants preferentially attended to relatively positive stimuli in the matrices. Alternatively, high-depression individuals were more even in their distribution of attention to positive and negative stimuli, reflecting what the authors referred to as a more “accurate” attention distribution. While we did not find evidence for a positive bias (e.g., avoidance of sad faces) amongst our low-depression participants, our more-depressed participants in the eye-tracking sample and one mouse-tracking sample had greater scan time for sad faces than neutral faces, consistent with Lazarov et al. [30] and Klawohn et al. [31].

Our study also extends previous work by demonstrating how negative attention bias can be assessed remotely using a task that captures the elaborative processing of emotional stimuli. Until recently, the measurement of attention bias has been restricted to reaction time-based tasks like the dot-probe or assessments that utilize eye-tracking data. While eye-tracking data used in combination with free viewing tasks seems to produce stronger attention bias metrics in depression, this data can be challenging and expensive to collect. We have shown that mouse-tracking can be used with an attention bias task to assess bias remotely without the need for expensive equipment. Further, because the task is free-viewing, it may capture more naturalistic (and elaborative) attention processes, which have been implicated as key mechanisms in depression [30,40]. This contrasts with anxiety, where attentional vigilance for threat-related stimuli is a key mechanism.

Two of our three samples supported evidence for a depression by stimuli valence interaction when predicting time spent viewing stimuli in the task. That is, for the eye tracking and one of the mouse-tracking studies, depression severity was associated with greater scan time for sad stimuli relative to neutral stimuli. At lower levels of depression, there was no difference between viewing time for sad and neutral stimuli. We intentionally included the null findings from sample 3 in an effort to avoid contributing to the “file drawer” predicament many null results find themselves in [69,70].

It is tempting to speculate which results are true findings and which are the result of a Type I or II error. Confirmatory bias often leads us to believe the significant effects and dismiss the non-significant findings as erroneous. However, thinking about results in a binary fashion misses the mark – even if all three samples yielded significant results, we really can only discuss in terms of degrees of evidence. That is, significant findings in three samples would increase our confidence in the effect; significant results in only two of those samples tempers that confidence. Including all three studies gives a more realistic estimation of the effect, which is better for the field.

Why might we not have observed these effects across all three samples? Our power analyses demonstrated that we were underpowered to detect smaller interaction effects in our remote samples. It is also possible that the effect demonstrated in Study 2 was the result of noise. Future work will need to collect data from relatively large samples to address these concerns. Moreover, our sample lacked symptom severity. Average BDI-II in study 1 was 9.1 (SD =  7.9), which falls in the “Minimal depression” range. In study 2, a mean depression score of 11.2 (SD =  6.4) on the PHQ-8 falls in the “moderate depression” range and an average of 9.9 (SD =  5.8) in study 3 constitutes “mild depression.” Therefore, while we see this as a promising first step by assessing the feasibility of assessing depression-related attention bias using a relatively new task, both in person and remotely, relatively few instances of people with high levels of depression may have made it more difficult to detect depression-related attention effects.

Additionally, another difference between samples was that the eye-tracking data was collected in-person in the lab, while the mouse-tracking samples were collected entirely online where participants could be more distracted. A poor testing environment could further increase measurement error in the mouse-tracking data, which in turn can make it more difficult to detect significant interactions and moderator effects [71]. This stresses the importance of testing the psychometrics of both mouse-tracking and eye-tracking task data in all applicable contexts (e.g., both remote and in-person) in which we hope to administer them in the future, and not assuming good psychometrics in one setting will apply to another [72].

The observed effects in this study are admittedly small. As our coefficients are measured in milliseconds (ms), each unit increase in depression scores yields a small increase in ms of viewing sad versus neutral stimuli. For instance, in the absence of depression (i.e., BDI-II score of 0), the difference in viewing time on a given trial between sad and neutral stimuli is not different from zero (14 ms). This difference expands to a difference of 78 ms per trial at minimal depression (BDI-II of 13), 107 ms at mild depression (BDI-II of 19), 152 ms at moderate (BDI-II of 28), and 211 ms at severe depression (BDI-II of 40). This means that over the course of an approximately 10-minute task, someone with severe depression will view sad stimuli for 11.8 seconds more on average than someone with no depression. We observe a similar pattern in the mouse-tracking task, with participants with severe depression symptoms (IDAS dysphoria =  40) spending 19.0 seconds longer on average viewing sad stimuli over neutral, relative to participants with no depression (IDAS dysphoria =  0).

This raises an important point: while these effects are small, they accumulate over the course of the task, and in real-world settings, over the course of the day. If individuals with elevated depression symptoms repeatedly have small windows of increased exposure to negative information throughout the day, and less exposure to neutral (or potentially even positive information), the cumulative effect of this bias can result in depressed persons attending to, thinking about, and taking in more negative information than someone with low levels of depression. Indeed, new work using eye tracking to capture participant gaze while viewing a manufactured news website page showed that high-depression participants spent more time looking at sad articles over positive ones, compared to participants with minimal depression [73]. While the snapshot of effects here is small, the combined impact of additional moments of biased attention to dysphoria information (both external and internal) may maintain negative mood states.

This raises a second important point, and exciting area for future research: the design and ecological validity of these tasks. While most attention bias research measures externally directed attention via visual attention to images, this is somewhat of a construct mismatch to how cognitive theory posits attention bias may maintain depression. While there may be biased processing of externally-directed attention, negatively biased attention toward one’s own thoughts, moods, and internal states are also thought to play a role in core features of depression like rumination [74]. Measuring biased attention towards internal information using more personalized “stimuli” may also yield larger effects. Future work should explore the development of this type of idiographic measurement for negative attention bias. Finally, our tasks were free-viewing tasks where participants were encouraged to view the stimuli however they wished in order to encourage naturalistic processing of the images. Future work should evaluate the effect of alternative instructions or objectives as part of the task.

Nevertheless, we believe the present study is a promising first step towards demonstrating that depression-related attention bias can be measured remotely using a free-viewing task that allows for more naturalistic processing. The use of mouse-tracking to evaluate attentional biases seems particularly appropriate for depression, given the theoretical importance of sustained processing for the maintenance of depression. Mouse-tracking may also allow researchers to collect larger and more representative samples, which would be an important step towards rigorously testing long-standing and influential theories of psychopathology, such as the cognitive model of depression.
